# Culturing and Mating of *Aspergillus fumigatus*

**DOI:** 10.1002/cpmc.87

**Published:** 2019-07-01

**Authors:** George D. Ashton, Paul S. Dyer

**Affiliations:** ^1^ School of Life Sciences University of Nottingham Nottingham United Kingdom

**Keywords:** ascospores, *Aspergillus fumigatus*, cleistothecia, mating, sexual reproduction

## Abstract

*Aspergillus fumigatus* is an opportunistic human fungal pathogen, capable of causing invasive aspergillosis in patients with compromised immune systems. The fungus was long considered a purely asexual organism. However, a sexual cycle was reported in 2009, with methods described to induce mating under laboratory conditions. The presence of a sexual cycle now offers a valuable tool for classical genetic analysis of the fungus, such as allowing determination of whether traits of interest are mono‐ or poly‐genic in nature. For example, the sexual cycle is currently being exploited to determine the genetic basis of traits of medical importance such as resistance to azole antifungals and virulence, and to characterize the genes involved. The sexual cycle can also be used to assess the possibility of gene flow between isolates. This is an open access article under the terms of the Creative Commons Attribution License, which permits use, distribution and reproduction in any medium, provided the original work is properly cited.

This unit describes protocols for culturing of *A. fumigatus* and for inducing sexual reproduction between compatible *MAT1‐1* and *MAT1‐2* isolates of the species. The unit also provides working methods for harvesting sexual structures, isolating single‐spore progeny and confirming whether sexual recombination has occurred. © The Authors. This is an open access article under the terms of the Creative Commons Attribution License, which permits use, distribution and reproduction in any medium, provided the original work is properly cited.

## INTRODUCTION

*Aspergillus fumigatus* is a very common environmental fungus, often isolated from air samples and from rotting vegetation. One reason for the success of the species is the ability to produce prolific numbers of asexual spores (Samson, Varga, & Dyer, 2009). These conidia are of relatively small size (2‐ to 3‐µm in diameter), which means that they can become easily airborne and wind dispersed. A complication is that the spores can also penetrate deep into the airways of humans and other animals, with up to two hundred spores estimated to be inhaled per day by the average person (Dagenais & Keller, 2009; Latgé, 2001). For most individuals the conidia will be removed by the host immune system. However, the fungus can trigger asthmatic reactions, and in those with suppressed immune systems the fungus can cause a range of diseases collectively termed “aspergillosis.” The most serious invasive forms have high mortality rates despite the use of antifungal drugs (Dagenais & Keller, 2009; Kwon‐Chung & Sugui, 2013). The medical treatment of disease has been complicated by the appearance and spread of resistance to azole antifungal drugs in *A. fumigatus*. This phenomenon was first reported in the late 1990s, and now perhaps 5% to 15% of clinical isolates are thought to be resistant to front‐line azoles (Abdolrasouli et al., 2018; Lockhart et al., 2011; van der Linden et al., 2015). The genetic basis of resistance has been determined in most, but not all, such isolates and most often involves sequence changes in the *cyp51A* target gene or its promoter region (Lestrade, Meis, Melchers, & Verweij, 2019; Rivero‐Menendez, Alastruey‐Izquierdo, Mellado, & Cuenca‐Estrell, 2016).

*Aspergillus fumigatus* has been considered a purely asexual organism for most of its described history. However, genomic, population and genetic analyses indicated the possibility of sexual reproduction in the species, with isolates of *MAT1‐1* and *MAT1‐2* genotype identified from a global sampling of the species (Paoletti et al., 2005). Such complementary *MAT1‐1* and *MAT1‐2* isolates are characteristic of sexually reproducing heterothallic (obligate outbreeding) ascomycete fungi including *Aspergillus* species, in which mating‐type identity is determined by the presence of *MAT1‐1* or *MAT1‐2* family mating‐type genes (Dyer, Inderbitzin, & Debuchy, 2016; Ojeda‐Lopez et al., 2018). A major breakthrough was then made in 2009, when O'Gorman and co‐authors reported the discovery of a teleomorph (sexual stage) of *A. fumigatus*, which was named *Neosartorya fumigata* (O'Gorman, Fuller, & Dyer, 2009). As part of the work, O'Gorman et al. 2009 described a method to induce sexual reproduction in *A. fumigatus* by crossing known *MAT1‐1* and *MAT1‐2* isolates on oatmeal agar from an apparently recombinant population from Dublin, Ireland. After a six‐month incubation period, sexual structures known as “cleistothecia” were formed containing sexual ascospores within asci. Evidence for sexual recombination in the progeny was provided by DNA fingerprint analysis (O'Gorman et al., 2009). Follow‐up work by Sugui et al. (2011) led to the identification of “supermater” strains of *A. fumigatus* that were able to undergo the full sexual cycle within a shorter time period of three months, with ascospores present even after 4 weeks in certain crosses, and which were able to form relatively high numbers of cleistothecia with a wide range of isolates.

This unit describes the protocol for inducing the sexual cycle of *A. fumigatus* under laboratory conditions by mating suitable strains, and methods for the collection and sorting of ascospore progeny, thereby providing the user with the tools to exploit this system for novel biomedical research.

*CAUTION: Aspergillus fumigatus* is a Biosafety Level 2 (BSL‐2) pathogen. Follow all appropriate guidelines and regulations for the use and handling of pathogenic microorganisms. See Current Protocols article (Coico & Lunn, 2006) and other pertinent resources (see Current Protocols article; 2006) for more information. Where open plate work is being performed suitable user protection must be applied, for example use of face mask and avoidance of air flow which might disperse conidia.

## STRATEGIC PLANNING

### Strain Acquisition

Experience has shown that isolates of *A. fumigatus* can vary greatly in their sexual fertility, and furthermore that prolonged sub‐culture by repeated asexual transfer can result in decreased or even total loss of sexual fertility (Houbraken & Dyer, 2015; Sugui et al., 2011). Therefore, it is important that as part of any crossing efforts, that ideally control crosses between known sexually fertile *MAT1‐1* and *MAT1‐2* reference strains are included to ensure that suitable conditions are present to induce the sexual cycle. The supermater strains AFB62 (=47‐267) (*MAT1‐1*) and AFIR928 (=47‐55) (MAT1‐2) of Sugui et al. 2011 together with a range of fertile Irish *MAT1‐1* and *MAT1‐2* strains from the study of O'Gorman et al. 2009 are available from the Fungal Genetics Stock Centre (USA), as well as on request from the authors concerned.

### Long‐Term Culture and Storage

*Aspergillus fumigatus* can be maintained through serial culture on a variety of solid media [e.g., *Aspergillus* complete medium (ACM; Paoletti et al., 2005) or Malt Extract Agar (MEA; see below)] on plates or slants with growth optimal between 25° to 37°C. To promote asexual sporulation, cultures should not be sealed for the first 5 days of incubation, but can be sealed appropriately after that stage, with cultures routinely stored up to 6 months at 4°C before re‐streaking as necessary. Ideally all stock cultures will be established from a single‐spore derived source. It is also important to establish long‐term frozen stocks. Typically, 10% glycerol stocks at −80°C will provide consistent viability for at least 5 years, whilst storage under liquid nitrogen preserves cultures nearly indefinitely. Given the risk of decreased sexual fertility following prolonged subculture, for critical work it is recommended to establish new crossing cultures from long‐term frozen stocks.

### Mating of *Aspergillus fumigatus*

The successful mating of *A. fumigatus* requires a series of co‐ordinated steps as will now be described.

## Basic Protocol 1 OATMEAL AGAR PREPARATION

In order to induce sexual reproduction in *A. fumigatus*, the complementary mating partners must be exposed to the correct growth conditions. Arguably the most important of which is the incubation of *A. fumigatus* on oatmeal agar (OMA), which provides the nutrients required for production of cleistothecia. Although commercially available, best results are obtained by preparing new batches of OMA from fresh materials. A protocol is shown below for the preparation of a 2‐litre batch of oatmeal agar.

### Materials

Tap water
80 g Irish pinhead oatmeal (Odlums)
40 g agar

5‐liter flask
Heat plate with magnetic stirrer
Aluminum foil
Two layers of cheese cloth
Four 1‐liter Schott bottles
Magnetic fleas
Autoclave for sterilization
9‐cm petri dishes

1 Add 2 liters of tap water to a 5‐liter flask.
2 Add 80 g Irish pinhead oatmeal (Odlums) to the flask.
3 Bring to the boil using a hotplate, stirring continuously with a magnetic flea to ensure the oatmeal does not burn.
4 Cover the top of the flask with foil to prevent water loss.
5 After the water has reached boiling temperature, lower the temperature of the hotplate so the mixture is bubbling gently and cook for 45 min—be careful that the oatmeal does not boil over.
6 Once cooked, filter the oatmeal solution through two layers of cheese cloth to remove the majority of solids. This will leave a cream‐colored viscous liquid. To ensure faster filtration, remove any solids that may build up in the cheese cloth.
7 Restore volume to 2 liters with tap water and mix the solution. Typically, 10% of the volume is lost during cooking.
8 To each 1‐liter Schott bottle add 10 g agar.
9 Add 500 ml of the oatmeal liquid into each bottle.
10 Make sure the oatmeal and agar have mixed thoroughly. To do this, place a magnetic flea inside each Schott bottle and stir for 5 min. Once mixed, remove the flea.
11 Autoclave the bottles, ensuring the lid is loosely sealed, for 30 min at 117°C, followed by ambient rather than forced cooling (this is essential to prevent boiling over of the viscous media from the bottles). Autoclaved media can be stored for up to 3 months.
12 Prior to crossing work, pour 25‐ml aliquots of oatmeal agar into 9‐cm petri dishes for each cross to be performed (ensuring each cross is performed in triplicate).
  From a 2‐liter batch of oatmeal agar, the user will be able to produce ∼80 oatmeal agar 9‐cm petri dishes. If more or less is required, adjust the quantities as needed. Note that in the authors’ experience, most consistent and reliable results are obtained using Irish pinhead oatmeal (Odlums, Ireland) to make the OMA, but that other sources of oatmeal (e.g., Quaker Oats) can also be used successfully.

## Basic Protocol 2 DETERMINATION OF MATING TYPE—MULTIPLEX PCR ASSAY

In order to perform a sexual cross between two isolates of *Aspergillus fumigatus*, two complementary mating partners of *MAT1‐1* and *MAT1‐2* genotype must be present due to the heterothallic nature of this species. It is therefore necessary to determine the mating type of an isolate of *A. fumigatus* prior to setting up crosses. For some isolates, genome information will be available, allowing screening for the presence of the *MAT1‐1* or *MAT1‐2* genes reported by Paoletti et al. 2005 which determine mating‐type identity [GenBank accession numbers AY898660 and AY898661 (*MAT1‐1*), and XM_749896 (*MAT1‐2*)]. Alternatively, a multiplex PCR assay can be performed to determine the mating type of the isolates, based on the protocol of Paoletti et al. 2005 as follows. For each strain of *A. fumigatus* used, DNA extraction can be performed following the guidelines of the Wizard genomic DNA purification kit (Promega).

### Materials

50 ng template DNA
Primers AFM1, AFM2, and AFM3
dNTPs
Red Hot *Taq* DNA Polymerase kit (Thermo Scientific) or another suitable enzyme
Nuclease‐free water
1.5% (w/v) agarose gel
1× Tris‐acetate‐EDTA buffer solution
Ethidium bromide
100‐bp DNA ladder
Wizard genomic DNA purification kit (Promega) or similar
Thermal cycler for PCR

1 The reaction volume for the multiplex mating‐type assay is 25 µl, containing: 50 ng template DNA of the *A. fumigatus* isolate being tested; 50 ng of primer AFM1; 50 ng of primer AFM2; 100 ng of primer AFM3; 200 µM of each dNTP; 1 U of Red Hot DNA Polymerase; 1× Red Hot DNA polymerase reaction buffer; and made up to 25 µl with nuclease‐free water. The details of the primers used are shown in Table 1.

**Table 1:** Primers for *Aspergillus fumigatus* Mating‐Type Assay (Paoletti et al., 2005)

Primer	Sequence	Target Region
AFM1	5′‐CCTTGACGCGATGGGGTGG‐3′	*MAT1‐1* idiomorph
AFM2	5′‐CGCTCCTCATCAGAACAACTCG‐3′	*MAT1‐2* idiomorph
AFM3	5′‐CGGAAATCTGATGTCGCCACG‐3′	‘Common’ flanking region

2 The PCR cycle parameters are as follows:

**Table:** 

1 cycle:	5 min	95°C (initial denaturation)
35 cycles:	30 sec	95°C (denaturation)
	30 sec	60°C (annealing)
	1 min	72°C (extension)
1 cycle:	5 min	72°C (final extension) (all at ramp rate 60°C min^−1^).
3 The products of this multiplex PCR assay can then be visualized using gel electrophoresis (1.5% w/v agarose gel in 1× Tris‐acetate‐EDTA buffer) stained with ethidium bromide (0.5 µl/ml). The amplicon size can then be estimated using a 100‐bp DNA ladder.
  The expected product size of MAT1‐1 isolates is 834 bp, while MAT1‐2 isolates have an amplicon size of 438 bp.

## Basic Protocol 3 CROSSING METHOD

Once mating type has been determined and two isolates of different mating type have been chosen for the sexual cross, the use of the following protocol for the inoculation of the two complimentary *A. fumigatus* isolates helps to ensure maximum number of cleistothecia are formed. Note that it is recommended to include a control cross between known fertile mating strains in addition to crosses with new test isolates.

### Materials

Spore suspensions (5 × 10^5^ conidia ml^−1^) of each isolate of *A. fumigatus*
Sterile 0.05% Tween 80
25 ml oatmeal agar 9‐cm Petri dishes (see Basic Protocol 1)

10‐µl inoculation loop
Hemacyometer or other suitable cell counter
2‐µl pipettes
Sterile pipette tips (use in steps 1 and 2 below)
Class II sterile hood (use in steps 1 to 3 below)
Parafilm (or Nescofilm) to seal petri dishes
30°C incubator in darkness

**Figure 1:**
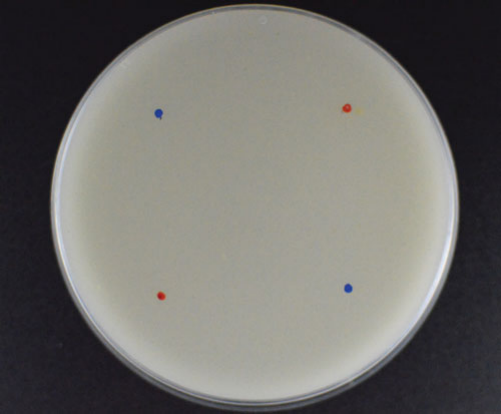
Oatmeal agar plates, marked with the points of inoculation. Blue for MAT1‐1 and red for MAT1‐2.

1 Prepare 1 ml spore suspensions of each isolate (5 × 10^5^ conidia ml^−1^) in 0.05% Tween 80 from 7‐ to 10‐day‐old grown cultures—using the 10‐µl inoculation loop and sterile 0.05% Tween 80 solution with spore counting by a hemacytometer method or similar (Aneja, 2003).
2 Inoculate two 1‐µl aliquots of each spore suspension (using a 2‐µl pipette) onto the oatmeal agar surface ∼4‐cm apart and perpendicular to aliquots of conidia of the opposite mating type. It is useful to keep a record of which mating partners are inoculated at the defined points, using for example red/blue marks or numbers (Fig. 1).

3 Seal inoculated petri dishes with one layer of Parafilm (or Nescofilm) and incubate inverted for 3 month at 30°C in complete darkness. Best results are obtained when plates are incubated in single depth rather than being stacked on top of each other.
4 Periodically examine the cultures over the course of 3 months to ensure the oatmeal agar does not dry out (resealing breaks in the Parafilm, as necessary).

## Basic Protocol 4 HARVESTING OF CLEISTOTHECIA

**Figure 2:**
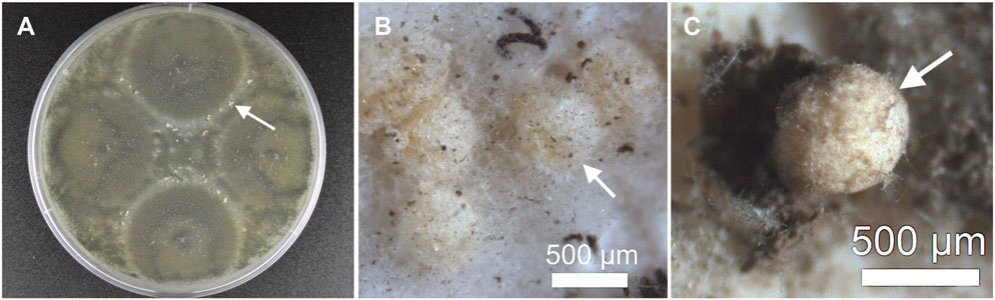
*Aspergillus fumigatus* sexual development. (**A**) Crossing plate following three months incubation at 30°C in darkness. Cleistothecia formation (white arrowed) can be observed at the barrage zones where the isolates of complementary mating type meet. (**B**) Group of cream‐colored cleistothecia of A. fumigatus (arrowed) covered by white mycelia, following removal of most superficial conidia by hoovering. (**C**) Individual cleistothecium of *A. fumigatus* (arrowed) following dissection from underlying tissues, ready for transfer for ascospore isolation.

After the 3‐month incubation period, examine each sexual cross for production of cleistothecia, visible as white‐cream colored hyphal spherical structures (150‐ to 6000µm diameter, darkening on maturity). Typically, these will appear at the barrage zones between two complementary mating partners, and may be scattered individually or group in small clusters (Figs. 2A‐C). These sexual structures contain the progeny from the *A. fumigatus* sexual cycle. Note that cleistothecia may be submerged under asexual conidiophores and hard to detect by eye. Inspection under a dissecting microscope is therefore often necessary to identify the cleistothecia, and the careful use of dissecting needles to tease apart tissues in the barrage zone can reveal the presence of cleistothecia that are not immediately apparent.

**Figure 3:**
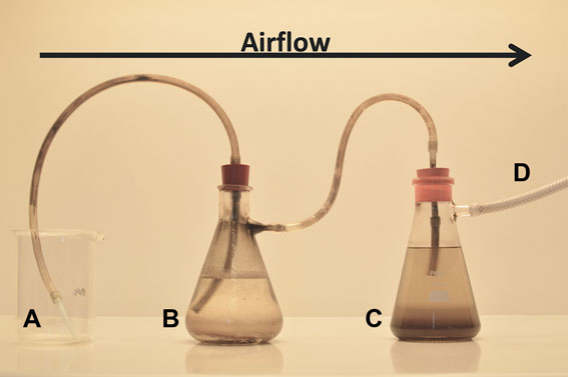
Image showing experimental set up for the ‘hoovering’ of conidia from *Aspergillus fumigatus* crossing plates. Note particularly: (**A**) A 1000‐µl pipette tip, allowing precise targeting of conidia; (**B**) Conical flask containing 2% trigene disinfectant, killing conidia that have been hoovered; (**C**) Conical flask containing isopropanol, killing any remaining conidia from (B); (**D**) Final tube connected to a 10‐µm filter (not shown), in order to trap any leftover conidia, leading to the vacuum tap. The airflow is in the direction of A‐D in all circumstances.

Furthermore, if it is necessary to accurately count the number of cleistothecia then the use of a “hoovering” technique is recommended (Sugui et al., 2011). This involves use of a blue sterile pipette tip (1000‐µl) linked to a vacuum line, with a trap vessel in between containing isopropanol together with 10 µm filter(s) to catch fungal material (Fig. 3). The pipette tip is carefully held above the barrage zone of the crossing plate, and the surface “hoovered” under gentle vacuum to remove conidia and hyphae obscuring the cleistothecia, taking care not to hoover up the cleistothecia themselves.

### Materials

0.05% Tween 80
Isopropanol for sterilization
Crossing plate containing cleistothecia (see Basic Protocol 3)
Sterile water
4% water agar plates
2% w/v malt extract agar (MEA; see recipe)

Class II sterile hood
Sterile 2‐ml microcentrifuge tubes
Fine‐point needle
Bunsen burner
Dissecting microscope
Vortex mixer
Heat block
28°C incubator in darkness

1 Transfer 100 µl sterile 0.05% Tween 80 into sterile 2‐ml microcentrifuge tubes.
2 Using a sterile fine‐point needle, sterilized using the isopropanol and Bunsen burner, isolate a cleistothecia from the crossing plate (from Basic Protocol 3). This is done most easily by observing cultures under a dissecting microscope when picking up cleistothecia.
3 Wash the cleistothecia of adhering conidia by rolling the cleistothecia through 20 µl sterile water on a 4% water agar plate.
4 Using a needle, transfer the washed cleistothecia into the 2‐ml microcentrifuge tube (from step 1), and crush the cleistothecia in the 100 µl of 0.05% Tween 80. Ideally 5 to 10 cleistothecia will be used at this stage, although a minimum of one cleistothecia is possible.
5 Adjust the volume of the 2‐ml microcentrifuge tube, adding 1.9 ml of 0.05% Tween 80.
6 Vortex the solution for 1 min in order to ensure all the ascospores have been released and asci sufficiently disrupted.
7 Heat for 1 hr at 70°C to kill remaining conidia and mycelial tissues.
8 During this time, pour 25 ml of 2% malt extract agar (MEA) plates ensuring enough are poured to allow for triplicates of each ascospore solution.
9 Spread plate aliquots of 100 µl of heat‐treated ascospores onto 2% (w/v) malt extract agar (MEA) plates and incubate for 36 hr at 28°C (use triplicate technical repeats of the ascospore suspension because the spread plated ascospores may be too dense or dilute on particular replicates).
  CAUTION: When isolating cleistothecia from A. fumigatus sexual crosses, wear a safety mask to minimize the risk of inhalation of conidia and avoid air currents that might cause conidia to become airborne.

## Basic Protocol 5 ISOLATION OF INDIVIDUAL ASCOSPORES

After 36 hr incubation, the ascospores will have begun germinating. This protocol will describe isolation procedures to enable single ascospore progeny to be isolated for further studies.

### Materials

Plate with germinating ascopores (see Basic Protocol 4)
Isopropanol for sterilization
25 ml 2% MEA plates

Class I sterile hood
Dissecting microscope
Fine‐point needle
Bunsen burner

1 Using a dissecting microscope within a class I sterile hood, identify individual germinating ascospores. See image (Fig. 2h) from O'Gorman et al. 2009 for an example.
2 Once single germinating ascospores have been identified, use a sterile fine‐point needle, sterilized using the isopropanol and Bunsen burner, to carefully cut out the single germinating ascospore. Alternatively, a LaRue lens cutter or similar single‐spore device may be used.
3 Place germinating ascospore on the center of a 25 ml 2% (w/v) MEA or ACM plate.
4 Incubate at 28°C in darkness or diffuse light until fully grown.

## Basic Protocol 6 CONFIRMATION OF RECOMBINATION—RAPD‐PCR ASSAY

Once individual ascospore progeny from the *A. fumigatus* have been isolated, it is often valuable to validate that sexual recombination has occurred. Various methods are available, but a relatively straightforward method is to perform DNA fingerprinting of the offspring using random amplification of polymorphic DNA (RAPD‐PCR), as described by Murtagh, Dyer, McClure, and Crittenden (1999). For each ascospore progeny of *A. fumigatus* used, DNA extraction can be performed following the guidelines of the Wizard genomic DNA purification kit (Promega).

### Materials

0.1 to 1 ng genomic DNA from each isolate ascospore
10 base‐pair RAPD primer
dNTPs
DyNAzyme II DNA polymerase kit (Thermo Scientific) containing:
  DyNAzyme II DNA polymerase
  1× DyNAzyme II DNA polymerase buffer
Nuclease‐free water
1.5% (w/v) agarose gel
10× Tris‐boric acid‐EDTA buffer solution
Ethidium bromide
1‐kb DNA ladder, optional
Wizard genomic DNA purification kit (Promega) or similar
Thermal cycler for PCR

1 The reaction volume for the RAPD‐PCR assay is 25 µl, containing: 0.1 to 1 ng template DNA from ascospore progeny (note the relatively low amount of DNA template); 50 µmol of a single 10‐base‐pair primer; 500 µM of each dNTP; 1 U of DyNAzyme II DNA polymerase; 1× DyNAzyme II DNA polymerase buffer; and adjusted to 25 µl with nuclease‐free water. Details of recommended primers for validation of *A. fumigatus* sexual recombination are shown Table 2.

**Table 2:** Primers for RAPD‐PCR Diagnostic

Primer	Sequence
A‐05	5′‐AGGGGTCTTG‐3′
J‐01	5′‐CCCGGCATAA‐3′
W‐10	5′‐TCGCATCCCT‐3′
W‐19	5′‐CAAAGCGCTC‐3′
X‐05	5′‐CCTTTCCCTC‐3′

2 The PCR cycle parameters are as follows:

**Table:** 

1 cycle:	5 min	95°C (initial denaturation)
45 cycles:	30 sec	93°C (denaturation; at a ramp rate of 30°C min^−1^)
	40 sec	37°C (annealing; at a ramp rate of 30°C min^−1^)
	1 min 20 sec	72°C (extension; at a ramp rate of 20°C min^−1^)
1 cycle:	5 min	72°C (final extension).
3 The products of the RAPD‐PCR assay can be visualized using gel electrophoresis (1.5% w/v agarose gel in 10×Tris‐boric acid‐EDTA buffer). Due to the high number of products that may be generated from the RAPD‐PCR, electrophoresis gels should be run for an extended period to ensure full resolution of products (approx. 2.5 to 4 hr). For this reason, the gel must be stained with ethidium bromide post‐electrophoresis. Depending on the primer used for the RAPD‐PCR assay, the expected products will vary and product sizes should be estimated using a 1 kb DNA ladder. The parental RAPD profiles are then compared to the progeny RAPD profiles.

## REAGENTS AND SOLUTIONS

### Ethylenediaminetetraacetic acid (EDTA), 0.5 M

181.1 g EDTA
600 ml H_2_O
Adjust to pH 8 with NaOH
Make up to 1 liter with dH_2_O
Autoclave for 15 min at 121°C
Store up to 6 months at room temperature

### Malt extract agar (MEA) 2%

20 g L^−1^ malt extract
6 g L^−1^ peptone
16 g L^−1^ agar
Autoclave for 15 min at 121°C
Store up to 3 months at room temperature

### Oatmeal agar (OA)

40 g L^−1^ Irish pinhead oatmeal
20 g L^−1^ agar
1 liter tap water
Store up to 3 months at room temperature

### Tris‐acetate‐EDTA buffer (TAE), 50×

242 g L^−1^ tris
57.1 ml glacial acetic acid
100 ml of 0.5 M EDTA (see recipe)
Make up to 1 liter with dH_2_O
Store up to 6 months at room temperature

### Tris−boric acid‐EDTA buffer (TBE), 10×

108 g L^−^ tris
55 g L^−1^ boric acid
20 ml of 0.5 M EDTA (see recipe)
Make up to 1 liter with dH_2_O
Store up to 6 months at room temperature

### TWEEN 80, 0.05%

500 µl TWEEN 80
Make up to 1 liter with dH_2_O
Store up to 3 months at room temperature

## COMMENTARY

### Background Information

The protocol described within this unit provides the user with sufficient knowledge to perform a sexual cross using complementary mating partners of *Aspergillus fumigatus*, based on methods developed by O'Gorman et al. 2009 and Sugui et al. 2011. There are several advantages to using the techniques discussed within this unit. Incubating the *A. fumigatus* crosses using the barrage zone method described in Basic Protocol 3, allows large areas of interaction between the mating partners, which helps to maximize the number of cleistothecia produced. Other crossing methods such as use of a mixed spore inoculum or spermatization (Houbraken & Dyer, 2015) have been attempted with *A. fumigatus*, but the barrage zone method has proved the most successful in terms of production of cleistothecia. Furthermore, the relatively high temperature resistance of ascospores means that adhering conidia and mycelial tissues can be eliminated by use of the extended 70°C heat shock for 1 hr, which is very advantageous to select for just sexual progeny. The presence of high‐temperature resistant ascospores is only seen in certain Sections of the *Aspergilli*, such as those with *Neosartorya* teleomorphs (Samson & Varga, 2010). The method described has been successfully implemented in independent laboratories in Ireland, the UK, Germany, the Netherlands and the USA to date (Camps et al., 2012; O'Gorman et al., 2009; Sugui et al., 2011; Szewczyk & Krappmann, 2010; Yu et al., 2017).

However, there are disadvantages to the techniques described above. Even using highly fertile reference strains, a minimum of three months is ideally required to ensure high numbers of mature cleistothecia. Use of increased external CO_2_ levels and alternative growth media might speed up sexual development (Dyer & O'Gorman, 2012). However, so far only oatmeal agar has been used reliably to induce the sexual cycle, although there are reports of formation of cleistothecia on composting waste (Zhang et al., 2017). Furthermore, mating conditions have been described as ‘fastidious’ (Kwon‐Chung & Sugui, 2009), with some research groups unable to induce sexual mating. In addition, validation of sexual recombination can be time consuming.

The sexual cycle in *A. fumigatus* nevertheless provides a powerful genetic tool to study previously uncharacterized biological processes, as this can be used to determine whether traits of interest are either mono‐ or poly‐genic in nature (Ashton & Dyer, 2016). For example, the sexual cycle has recently been exploited for medical purposes to determine the genetic basis of resistance to azole antifungals in *A. fumigatus*. The sexual cycle also allows the implementation of powerful techniques such as bulk segregant analysis (BSA) and quantitative trait loci (QTL) to identify defined genes which contribute to a particular trait.

### Critical Parameters and Troubleshooting

There are various critical components to ensure success of sexual mating in *A. fumigatus*. First, the use of control strains of known fertility which have not been subject to extended vegetative sub culture is recommended. Second, the use of triplicate (or more) repeat plates for each sexual cross is recommended as there can be inherent variability between sexual crossing plates. Third, periodic checking of the sexual crosses during incubation at 30°C is essential for success. If the sealing film used to seal the plates breaks during the three‐month incubation period, the oatmeal agar will dry out and result in a failed sexual cross. Ideally plates are also incubated inverted without stacking in the incubator.

If sexual development fails to occur when the user performs a sexual cross, this may be due to a number of reasons:

1. The oatmeal agar may have been prepared incorrectly. If this is the case, repeat experiment. Note that the recipe uses tap water, and that some local supplies might lack certain mineral nutrients, such as manganese or phosphorus, that have elsewhere shown to be required for sexual development in *Aspergillus* species (Dyer & O'Gorman, 2012).
2. The two mating partners may be unable to complete a sexual cycle together, regardless of being complementary *MAT1‐1* and *MAT1‐2* mating types. This might be due to factors such as loss of fertility due to extended vegetative subculture and/or inherent low fertility of certain crosses arising from isolates being of different clades and vegetative compatibility groupings of *A. fumigatus* (Ashu, Hageb, Chowdhary, Meis, & Xu, 2017; Dyer, Ingram, & Johnstone, 1992).
3. The incubator used might not provide adequate stable conditions for sexual development, such as constant temperature and humidity. Highly reliable incubators produced by Memmert (Germany) are used in the author's laboratory.
4. Some sexual crosses might exceptionally require more than 3 months for completion. It is recommended to incubate crossing plates for at least 6 months in the dark at 30°C before disposal of plates, to allow for possible delayed/late development of cleistothecia.

### Understanding Results

After performing the protocol described in this unit, the user should obtain a number of cleistothecia from which ascospores can be isolated.

#### Determination of mating type

The multiplex mating‐type assay is expected to yield PCR product sizes of 834 bp if the isolate is a *MAT1‐1* genotype and 438 bp if the isolate is a *MAT1‐2* genotype.

#### Harvesting of cleistothecia

The user should expect cleistothecia production primarily at the barrage zone of each sexual cross. Cleistothecia range in size from ∼200 to 600 µm and appear initially a white color, then becoming cream to yellow on aging. If mature, it should be possible to easily pick these sexual structures from the sexual cross using a fine point needle. However, it is important to stress that cleistothecia, even if present, might be hard to detect as they can be immersed under layers of asexual conidiophores. Therefore, the use of a dissecting microscope is needed to detect cleistothecia and judicious use of dissecting needles to tease apart the area of the barrage zone, together with hoovering of the surface of crossing plates (if necessary) as described above.

#### Isolation of individual ascospores

After 36 hr incubation of the ascospore solution, the user should expect to see germinating fungal colonies on the 2% MEA plate. Once the individual ascospores have been isolated, it should be straightforward to establish a single‐spore derived fungal culture.

#### Determination of recombination

If sexual recombination has occurred, the RAPD‐PCR profiles will be expected to show a mixture of PCR products in the progeny derived from the fingerprint patterns seen in the RAPD‐PCR profiles of the two parents.

### Time Considerations

The time considerations of this unit vary throughout each protocol depending on how many sexual crosses the user wishes to perform and how many samples are isolated from the germinating ascospores. However, the 3‐month incubation time of the *A. fumigatus* crosses must be considered as that is the most time‐consuming protocol within the unit. Generally, the total time to perform the whole unit is approximately 3.5 months, although some crosses can exceptionally require incubation for at least 6 months.
